# Keap1-targeting microRNA-941 protects endometrial cells from oxygen and glucose deprivation-re-oxygenation via activation of Nrf2 signaling

**DOI:** 10.1186/s12964-020-0526-0

**Published:** 2020-02-26

**Authors:** Shu-ping Li, Wei-nan Cheng, Ya Li, Hong-bin Xu, Hui Han, Ping Li, Deng-Xia Zhang

**Affiliations:** 1 Obstetrics and Gynecology Department, the Affiliated Changzhou No. 2 People’s Hospital of Nanjing Medical University, No. 188 Gehu Lake Road, Wujin District, Changzhou, Jiangsu China; 2 Department of Orthopedics, The First Affiliated Hospital of Xiamen University, Xiamen, China; 3 The Central Lab, North District, Suzhou Municipal Hospital affiliated to Nanjing Medical University, Suzhou, China; 4 Department of Radiotherapy and Oncology, Affiliated Kunshan Hospital of Jiangsu University, Suzhou, China

**Keywords:** microRNA-941, Nrf2, Keap1, Endometrial cells, Ischemia-reperfusion injury

## Abstract

**Background:**

Mimicking ischemia-reperfusion injury, oxygen and glucose deprivation (OGD)-re-oxygenation (OGDR) applied to endometrial cells produces significant oxidative stress and programmed necrosis, which can be inhibited by nuclear-factor-E2-related factor 2 (Nrf2) signaling. MicroRNA (miRNA)-induced repression of Keap1, a Nrf2 suppressor protein that facilitates Nrf2 degradation, is novel strategy to activate Nrf2 cascade.

**Methods:**

MicroRNA-941 (miR-941) was exogenously expressed in HESC and primary human endometrial cells, and the Nrf2 pathway examined by Western blotting and real-time quantitative PCR analysis. The endometrial cells were treated with OGDR, cell programmed necrosis and apoptosis were tested.

**Results:**

MiR-941 is a novel Keap1-targeting miRNA that regulates Nrf2 activity. In T-HESC cells and primary human endometrial cells, ectopic overexpression of miR-941 suppressed Keap1 3′-UTR (untranslated region) expression and downregulated its mRNA/protein expression, leading to activation of the Nrf2 cascade. Conversely, inhibition of miR-941 elevated Keap1 expression and activity in endometrial cells, resulting in suppression of Nrf2 activation. MiR-941 overexpression in endometrial cells attenuated OGDR-induced oxidative stress and programmed necrosis, whereas miR-941 inhibition enhanced oxidative stress and programmed necrosis. MiR-941 overexpression and inhibition were completely ineffective in Keap1−/Nrf2-KO T-HESC cells (using CRISPR/Cas9 strategy). Restoring Keap1 expression, using an UTR-depleted Keap1 construct, abolished miR-941-induced anti-OGDR activity in T-HESC cells. Thus Keap1-Nrf2 cascade activation is required for miR-941-induced endometrial cell protection.

**Conclusions:**

Targeting Keap1 by miR-941 activates Nrf2 cascade to protect human endometrial cells from OGDR-induced oxidative stress and programmed necrosis.

Video Abstract

**Graphical abstract:**

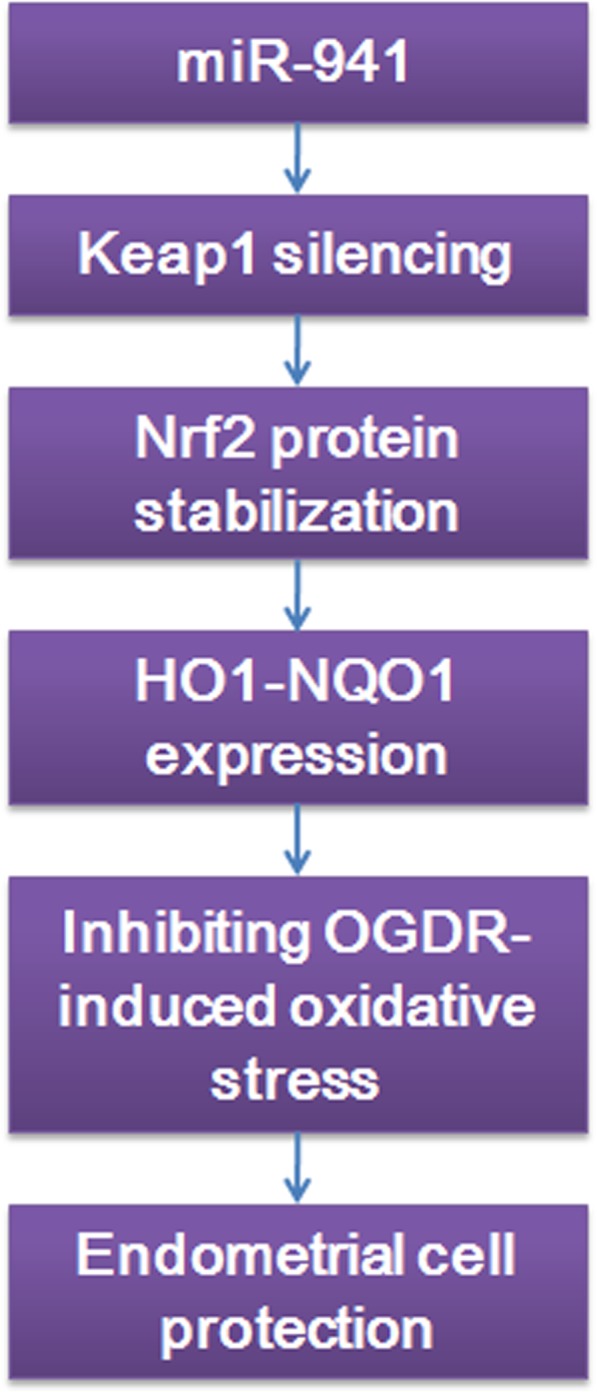

## Background

Postpartum hemorrhage is one of the most common complications in obstetric practice [1–3], resulting in ischemia to the endometrium and necrosis of endometrial cells [1–3]. To study the pathophysiology of ischemic death [4, 5], the oxygen and glucose deprivation (OGD)-re-oxygenation (OGDR) model is a useful approach to investigate ischemic-reperfusion endometrial cell injury [6–9]. Following reperfusion, endometrial ischemia induces oxidative stress to the endometrial cells [1–3], leading to the accumulation of reactive oxygen species (ROS) and depletion of antioxidants [10–12]. Oxidative injury causes excessive DNA breaks, protein damage and mitochondrial dysfunction [10–12], eventually leading to endometrial cell necrosis [1–3].

It has been recently shown that cell necrosis can occur via a programmed cell death process [13–16], called “programmed necrosis”. In endometrial cells, OGDR promotes p53 translocation to the mitochondria where it forms a complex with the mitochondria permeability transition pore (mPTP) component proteins, cyclophilin-D (CypD) and adenine nucleotide translocator type 1 (ANT1) [17, 18]. This initiates mitochondrial depolarization, mPTP pore opening and cytochrome C release to cytosol, eventually leading to cell necrosis, but not apoptosis [13–15, 19–22]. We previously reported that CypD inhibition or silencing (by targeted shRNA) suppressed OGDR-induced cytotoxicity and programmed necrosis in endometrial cells [5]. Ginseng Rh2 (GRh2) and keratinocyte growth factor (KGF) also protected endometrial cells from OGDR by shutting down the programmed necrosis pathway [4, 5].

The transcription factor nuclear-factor-E2-related factor 2 (Nrf2) promotes the expression of multiple key antioxidant genes and detoxifying enzymes, protecting cells against oxidative injury [23–25]. Nrf2 is localized to the cytoplasm where it binds Kelch-like ECH-associated protein 1 (Keap1) [23–25] and Cul3 ubiquitin ligase, which promotes Nrf2 ubiquitination and proteasomal degradation [23–25]. Activated Nrf2 (i.e. via post-translational modifications) is released from the Keap1-Cul3 complex, leading to Nrf2 protein stabilization [23–25]. Subsequently, Nrf2 translocates to cell nuclei where it binds the antioxidant responsive element (ARE) [23–25], leading to transcription of Nrf2-dependent antioxidant genes [26–28]. The majority of the Nrf2-dependent genes, including *heme oxygenase-1 (HO-1)*, *NAD(P)H:quinone oxidoreductase 1 (NQO1)*, *γ-glutamyl cysteine ligase catalytic subunit* (*GCLC*) [23], exert potent antioxidant activity [29, 30].

Studies show that forced Nrf2 activation in endometrial cells enhances antioxidant activity and mitigates OGDR-induced oxidative injury. We have previously reported that KGF-induced Nrf2 activation efficiently protected endometrial cells from OGDR [4]. MicroRNAs (miRNAs) are essential and novel players in regulating activation of the Keap1-Nrf2 pathway [31–36]. These ~ 22-nt long single-strand non-coding RNAs (ncRNAs) bind to the 3′-untranslated region (3′-UTR) of the targeted mRNAs, causing degradation and/or translation repression [37, 38]. A potential strategy to activate the Nrf2 signaling cascade is to reduce Keap1 protein levels by expressing Keap1-targeting miRNAs [31, 35, 36]. Here we show that microRNA-941 (“miR-941”) targets Keap1 to activate Nrf2 and protects endometrial cells from OGDR-induced cytotoxicity.

## Materials and methods

### Chemical and reagents

Puromycin and ploybrene were purchased from Sigma-Aldrich Chemicals Co. (St Louis, Mo). The fluorescent dye JC-1 was provided by Invitrogen Thermo-Fisher (Shanghai, China). All cell culture reagents and qPCR reagents were obtained from Gibco BRL Co. (Grand Island, NY). The antibodies of the present study were provided by Santa Cruz Biotechnology (Santa Cruz, CA) and Cell Signaling Tech (Suzhou, China). The primers, sequences, constructs and virus were designed and provided by Shanghai Genechem Co. (Shanghai, China), unless otherwise motioned.

### T-HESC cell culture

As described previously [4, 5], the immortalized human endometrial cell line, T-HESC [39], was cultured in regular DMEM/Hams F-12 nutrient medium plus 10% FBS.

### Culture of primary human endometrial cells

The fresh human endometrial tissues, acquired from a written-informed consent uterine-bleeding patient (31-year old, administrated at Changzhou Second People’s Hospital, undergoing the partial hysterectomy surgery) were digested with 0.15% trypsin-EDTA (Sigma) and Collagenase I (Sigma) for 1 h, and then transferred to DMEM/Hams F-12 nutrient medium plus 15% FBS. Tissues were dissolved. Blood vessel cells, immune cells and other non-endometrial cells were abandoned through gravity sedimentation. The remaining human endometrial cells were resuspended and cultured in complete DMEM medium [5]. The primary human endometrial cells at passage 3–10 were utilized for biomedical assays. The protocols of using human tissues and cells were approved by the Ethics Committee at Changzhou Second People’s Hospital.

### miR-941 overexpression or inhibition

The pre-miR-941 nucleotide sequence and its anti-sense sequence were synthesized and sequence-verified by Shanghai Genechem Co. Each of the two was ligated to the GV248 construct (Shanghai Genechem Co.). The construct, along with the lentivirus-packing helper plasmids (psPAX2 and pMD2.G [33]), were co-transfected to HEK-293 T cells, forming the pre-miR-941-expressing lentivirus (“lv-pre-miR-941”) and the pre-miR-941 anti-sense lentivirus (“antagomiR-941”). Virus were enriched, filtered, and added directly to human endometrial cells (in the polybrene-containing medium). When necessary puromycin (5.0 μg/mL) was included in the medium to select stable cells, with miR-941 levels examined by qPCR.

### qPCR

The human endometrial cells, with the applied treatments, were harvest and the total cellular RNA extracted using TRIzol protocol [5]. We utilized an ABI Prism 7500 Fast Real-Time PCR system to carry out the quantitative real time-PCR (qPCR) assay. To calculate product melting temperature we applied the melt curve analyses. *Glyceraldehyde-3-phosphatedehydrogenase* (*GAPDH)* was always examined as the reference gene and the internal control, and the 2^−∆∆*C*t^ method utilized for the quantification of targeted mRNAs. The following mRNA primers were utilized: *NQO1* (NM_000903) forward, 5′-CATTCTGAAAGGCTGGTTTG and reverse, 5′-GGCTGCTTGGAGCAAAATAC; *HO1* (NM_002133) forward, 5′-GCTACCTGGGTGACCTGTCT and reverse, 5′-GGGCAGAATCTTGCACTTTG; *Nrf2* (NM_006164) forward, 5′-TGAGCATGCTTCCCATGAT and reverse, 5′-CTTCTCTAGCCGCTCTGTGG; *GAPDH* (NM_002046) forward, 5′-CGGAGTCAACGGATTTGGTCGTAT and reverse, 5′-AGCCTTCTCCATGGTGGTGAAGAC. *Keap1* (NM_203500) forward: 5′-TACGATGTGGAAACAGAGACGTGGA and reverse 5′-TCAACAGGTACAGTTCTGGTCAATCT. The primers cover exon junction/s, and the amplicons around 90–200 bp. miR-941 was normalized to U6. miR-941 and U6 primers were obtained from OriGene (Beijing, China).

### Keap1 3′-UTR activity

Keap1 3′-UTR reporter plasmid (containing the miR-941-binding sites, at position of 276–283) was generated using the same protocol described previously [31], which was transfected to human endometrial cells using the Lipofectamine 2000 protocol. Afterwards, cells were subjected to the applied genetic modifications, with the Keap1 3′-UTR luciferase activity tested through the Promega kit [40].

### Transfection of miR-941 mimic

Human endometrial cells were seeded into the six-well tissue culture plates (at 1 × 10^5^ cells in each well). Lipofectamine 2000 was utilized for the transfection of 500 nM of the wild-type (“WT”) or the mutant (“Mut”) miR-941 mimics (synthesized by Shanghai Genechem Co.). After 48 h, miR-941 levels were determined by qPCR.

### RNA-pull down assay

The RNA-Pull down assay was carried out through the previously-described protocol [41, 42], testing miR-941-bound mRNA using the Pierce Magnetic RNA Pull-Down Kit, Shanghai, China). In brief, T-HESC cells were transfected with biotinylated miR-941 mimic or control mimic (100 nmol/L) for 48 h, and cells were harvested using the lysis buffer described early [42]. The biotin-captured RNA complex was pulled down by incubating the cell lysates (600 μg of each treatment) with the streptavidin-coated magnetic beads [41]. The bound mRNA was purified using the RNeasy Mini Kit (QIAGEN, Shanghai, China), with expression of *Keap1 mRNA*, in the bound fractions examined by qPCR. Its levels were normalized to the input controls.

### Cell viability

Human endometrial cells were seeded into the 96-well tissue culture plates (3000 cells in each well). Following the applied treatments, a cell counting kit-8 (CCK-8) kit (Dojindo Laboratories, Kumamoto, Japan) was utilized to examine cell viability [5], with CCK-8 optic density (OD) examined at test-wavelength of 450 nm.

### Lactate dehydrogenase (LDH) assay

The human endometrial cells were seeded into the 12-well tissue culture plates (at 0.5 × 10^5^ cells in each well). LDH release to the cell medium, the quantitative marker of cell necrosis [43], was tested through a two-step LDH detection kit (Promega, Shanghai, China) [5]. LDH contents in the medium were always normalized to total LDH levels.

### OGD/re-oxygenation (OGDR)

As described previously [4, 5], human endometrial cells with applied genetic treatments were initially placed into an airtight chamber (95% N_2_/5% CO_2_) for 4 h, mimicking OGD. Thereafter, human endometrial cells were returned back to the complete medium and re-oxygenated. Cells were further cultured for applied time periods.

### Western blotting

Human endometrial cells were seeded into the six-well tissue culture plates (at 1 × 10^5^ cells in each well). Following the applied treatments, cellular lysates were achieved and quantified [4, 5]. The lysates proteins (40 μg per treatment into each lane) were separated by SDS-PAGE gels, and transferring to polyvinylidene difluoride (PVDF) blots [44]. The detailed protocols for Western blotting and data quantification (through the Image J software) were previously described [45, 46]. Assaying of nuclear fraction proteins was described early [46].

### Mitochondrial immunoprecipitation (Mito-IP)

T-HESC cells were harvested and resuspended [5], with the supernatants collected as the cytosolic fraction lysates. The pellets were resuspended to achieve mitochondrial fraction lysates and quantified [47]. Mitochondrial fraction lysates (300 μg per treatment) were pre-cleared (using anti-IgG Sepharose beads), and incubated with anti-CypD antibody (Santa Cruz Biotech). The mitochondrial complex was captured by anti-IgG. CypD-p53-ANT1 association was examined by Western blotting assaying of CypD-bound proteins.

### JC-1 mitochondrial depolarization assay

The human endometrial cells were seeded into the 12-well tissue culture plates (at 0.5 × 10^5^ cells in each well). In OGDR-stimulated cells with mitochondrial depolarization (“∆Ψ”) the JC-1 fluorescent dye shall aggregate, forming green monomers [48]. The detailed protocol of JC-1 protocol was discussed previously [5]. The JC-1 fluorescence intensity was examined at 530 nm (Titertek Fluoroscan, Germany). The representative JC-1 images, integrating both green and red fluorescence images, were also presented.

### Superoxide detection

Endometrial cells with the applied genetic treatments were initially seeded onto 96-well tissue-culturing plates (at 3 × 10^3^ cells of each well). Following the applied OGDR stimulation, the superoxide colorimetric assay kit (BioVision, Shanghai, China) was applied to examine the cellular superoxide contents. In brief, the superoxide detection reagent (50 μL/well) was added for 15 min under the dark, with the superoxide’s absorbance tested at the test-wavelength of 450 nm [31].

### Lipid peroxidation assay

As reported [31] endometrial cells with the applied genetic treatments were initially seeded onto the six-well tissue-culturing plates (at 1 × 10^5^ cells per well). Following the applied OGDR stimulation, the lipid peroxidation assay kit (Abcam, Shanghai, China) was utilized to examine cellular lipid peroxidation levels, via the malondialdehyde method. The lipid peroxidation levels, determined by the thiobarbituric acid reactive concentration, were tested and quantified using the previously-described protocol [31, 49].

### NQO1 activity

The detailed protocol for testing the relative NQO1 activity in human endometrial cells has been described elsewhere [50]. In brief, the inducer potency was quantified by using the NQO1 bioassay. T-HESC cells or the primary human endometrial cells (10^4^ per well of a 96-well plate), with applied genetic treatments, were cultured for 24 h. The NQO1 enzyme activity was tested and quantified in cell lysates using menadione as the substrate.

### Nrf2 or Keap1 knockout (KO)

The lentiCRISPR-Nrf2-KO-puro construct and the lentiCRISPR-Keap1-KO-puro construct were described early [31], each was transduced to T-HESC cells (cultured in the polybrene-containing complete medium). Nrf2 sgRNA (Target DNA Sequence: TACACATTCAGCTGGCGCGT, PAM Sequence: AGG) and Keap1 sgRNA (Target DNA Sequence: GTACGCCTCCACTGAGTGCA, PAM Sequence: AGG) were utilized. The GFP-positive T-HESC cells were sorted via FACS. The selected single cells were further incubated in complete medium with puromycin for 10 days, with Nrf2/Keap1 KO verified by Western blotting and/or qPCR assays.

### Keap1 re-expression

The Keap1 (with no 3′-UTR region) expression GV248 construct was designed and provided by Shanghai Genechem, transduced to T-HESC cells with miR-941 overexpression. Cells were then selected by puromycin for 10 days, with Keap1 re-expression verified by qPCR and Western blotting assays.

### Statistical analysis

Data were presented as mean ± standard deviation (SD). The repeated-measures analysis of variance (RMANOVA) with Dunnett’s post hoc test for multiple comparisons (SPSS 16.0, SPSS Co. Chicago, CA) were utilized to evaluate statistical significance. The two-tailed unpaired T test (Excel 2013) was carried out to examine significance between two specific treatment groups. *P* < 0.05 was considered statistically significant.

## Results

### miR-941 targets Keap1 and activates Nrf2 signaling in human endometrial cells

**Fig. 1:**
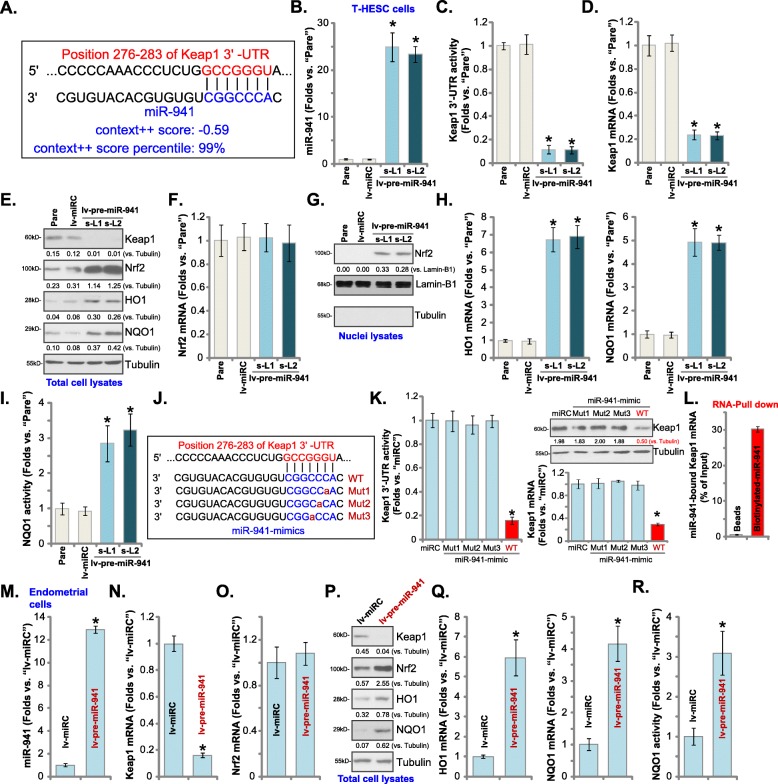
miR-941 targets Keap1 and activates Nrf2 signaling in human endometrial cells. miRNA-941 putatively targets the 3′-UTR (untranslated region) of human *Keap1 mRNA* (at position of 276–283) (**a**). T-HESC human endometrial cells were transduced with lentiviral pre-microRNA-941 (“lv-pre-miR-941”), with selection by puromycin the stable cells were established, with control cells transduced with lentiviral non-sense microRNA (“lv-miRC”); Expression of mature miR-941 and listed mRNAs was tested by qPCR assays (**b**, **d**, **f** and **h**); Keap1 3′-UTR activity was shown (**c**), with expression of listed proteins in total cell lysates (**e**) and nuclei lysates (**g**) tested by Western blotting; The relative NQO1 activity was tested as well (**i**). T-HESC cells were transfected with 500 nM of non-sense microRNA control (“miRC”), the wild-type (“WT”) or the mutant miR-941 mimics (sequences listed in **j**), with Keap1 3′-UTR activity (**k**) and Keap1 mRNA/protein expression (**k**) tested after 48 h. RNA-Pull down assay confirmed the direct association between biotinylated-miR-941 and *Keap1 mRNA* in T-HESC cells (**l**). The primary human endometrial cells (“Endometrial cells”, same for all Figures) were infected with lv-pre-miR-941 or lv-miRC, with expression of listed genes tested by qPCR (**m**-**o**, and **q**) and Western blotting (**p**) assays after 48 h. The relative NQO1 activity was tested as well (**r**). Expression of listed protein was quantified and normalized (**e**, **g, k** and **p**). “Pare” stands for the parental control cells (same for all Figures). Data were presented as mean ± SD (*n* = 5), and results were normalized. * ***P*** < 0.05 vs. “lv-miRC”/“miRC” cells. Experiments in this figure were repeated five times with similar results obtained

To identify Keap1-targeting miRNAs the TargetScan (V7.2, http://targetscan.org/) [51] database was first searched. Several potential miRNAs binding to 3′-UTR of Keap1 were identified and further examined using other miRNA databases, including miRbase (http://www.mirbase.org/) and miRDB (http://www.mirdb.org/miRDB/policy.html). The prediction algorithms identified miR-941 as a promising candidate that targets the 3′-UTR of Keap1 (at position of 276–283), with context^++^ score: − 0.59 and the score percentage of 99% (from TargetScan V7.2 [51], Fig. 1a). Functional studies were performed to validate whether miR-941 could affect Keap1. T-HESC human endometrial cells [4, 5] were transduced with a lentivirus encoding the pre-microRNA-941 (“lv-pre-miR-941”) and two stable cell lines, “s-L1” and “s-L2”, were established. Using qPCR [52], miR-941 levels increased over 20 folds in the lv-pre-miR-941-expresing stable cells (Fig. 1b). Significantly, Keap1 mRNA (Fig. 1d) and protein (Fig. 1e) were significantly downregulated, decreasing over 90% in miR-941-overexpressed cells (Fig. 1c). These results suggest that miR-941 selectively targets and downregulates Keap1 in endometrial T-HESC cells.

Testing the potential effect of miR-941 on Nrf2 signaling, we found that although Nrf2 mRNA levels (Fig. 1f) were unchanged in lv-pre-miR-941-expresing cells, Nrf2 protein levels (Fig. 1e) were significantly elevated (vs. control cells). Additionally, the stabilized Nrf2 protein translocated to T-HESC cell nuclei (testing nuclear lysates, Fig. 1g). Furthermore, mRNA and protein expression of two key Nrf2-dependent genes, including *HO1* and *NQO1* [33, 53–55], were significantly increased in miR-941-overexpressing cells (Fig. 1e and h), with the NQO1 activity significantly boosted (Fig. 1i). These results show that miR-941 overexpression can induce the Nrf2 signaling cascade activation in T-HESC cells.

To further validate that miR-941 targets Keap1, we transfected into T-HESC endometrial cells with three miR-941miRNAs with mutations in the binding sequence to the Keap1 3′-UTR, “Mut1/2/3” (sequences listed in Fig. 1j). As shown only the wild-type (WT) miR-941 decreased Keap1 3-UTR activity (Fig. 1k, the left panel) and *Keap1 mRNA*/protein expression (Fig. 1k, the right panel), while the miR-941 mutants were ineffective (Fig. 1k). These results further support that miR-941 targets *Keap1 mRNA* in T-HESC cells. The nonsense control miRNA (“miRC”) exerted no significant effect on Keap1 expression and Nrf2 signaling in T-HESC cells (Fig. 1b-k). To further support our hypothesis the RNA-Pull down assay was performed. As shown (Fig. 1l), biotinylated-miR-941 directly associated with Keap1 mRNA (Fig. 1 l).

In the primary human endometrial cells (“Endometrial cells”), infection with lv-pre-miR-941 led to an over 10 fold increase in the expression of mature miR-941 (Fig. 1m), causing downregulation of *Keap1 mRNA* (Fig. 1n). MiR-941 overexpression similarly induced Nrf2 signaling activation, leading to Nrf2 protein (but not mRNA) accumulation (Fig. 1o and p), HO1 and NQO1 upregulation (both mRNA and protein, Fig. 1p and q) as well as an increase in NQO1 activity (Fig. 1r). Collectively, these results show that miR-941 targets Keap1 and activates Nrf2 signaling in human endometrial cells.

### miR-941 overexpression protects human endometrial cells from OGDR

**Fig. 2:**
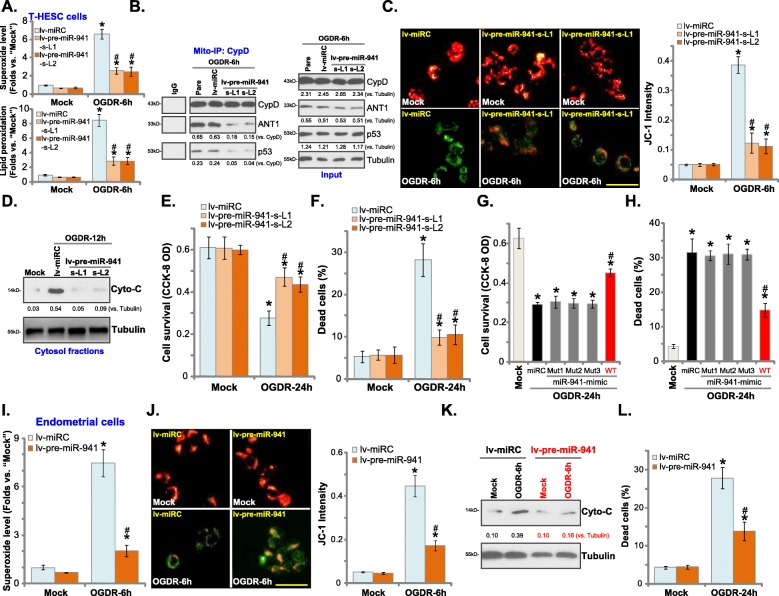
miR-941 overexpression protects human endometrial cells from OGDR. Stable T-HESC cells with lentiviral pre-microRNA-941 (“lv-pre-miR-941”, two lines) or the lentiviral non-sense microRNA (“lv-miRC”) were subjected to OGD exposure for 4 h, followed by re-oxygenation (“OGDR”) for applied time periods, ROS production (superoxide and lipid peroxidation contents, **a**), mitochondrial CypD-ANT1-p53 association (tested by mito-IP assay, **b**) as well as mitochondrial depolarization (JC-1 green fluorescence accumulation, **c**) and cytochrome C release (**d**, testing cytosol proteins) were tested; Cell survival and necrosis were tested by CCK-8 assay (**e**) and LDH release assay (**f**), respectively. T-HESC cells were transfected with 500 nM of non-sense microRNA control (“miRC”), the wild-type (“WT”) or the mutant miR-941 mimics for 48 h, followed by the same OGDR stimulation for another 24 h, cell survival (**g**) and necrosis (**h**) were tested. The primary human endometrial cells, with lv-pre-miR-941 or lv-miRC, were subjected to the same OGDR procedure, ROS production (superoxide contents, **i**), mitochondrial depolarization (**j**) and cytosol cytochrome C release (**k**) were tested similarly, with cell necrosis tested by LDH release assay (**l**). For mito-IP assay, CypD-bound ANT1 and p53 were quantified (**b**), with total levels of CypD, ANT1 and p53 tested as the “Input” control (**b**). For the cytochrome C release assay, relative cytosol cytochrome C level was quantified (**d** and **k**). Data were presented as mean ± SD (*n* = 5), and results were normalized. “Mock” stands for non-OGDR treatment (same for all Figures). * ***P*** < 0.05 vs. “Mock” treatment in “lv-miRC”/“miRC” cells. ^**#**^***P*** < 0.05 vs. OGDR treatment in “lv-miRC”/“miRC” cells. Experiments in this figure were repeated five times with similar results obtained. Bar = 50 μm (**c** and **j**)

Our previous studies [4, 5] have shown that OGDR mainly induced programmed necrosis, but not apoptosis, in endometrial cells. Here in the control T-HESC cells (with lv-miRC), OGDR similarly induced programmed necrosis, leading to superoxide accumulation and lipid peroxidation (Fig. 2a), mitochondrial CypD-ANT1-p53 association (Fig. 2b), mitochondrial depolarization (tested by JC-1 green fluorescence accumulation, Fig. 2c) and cytosol cytochrome C release (Fig. 2d). Significantly, ectopic overexpression of miR-941, using lv-pre-miR-941, potently inhibited OGDR-induced oxidative stress and programmed necrosis in T-HESC cells (Fig. 2a-d). The lv-pre-miR-941-expressing T-HESC cells were protected from OGDR, as indicated by increased cell viability (Fig. 2e) and reduced necrosis (medium LDH release, Fig. 2f), when compared to control cells. Further, T-HESC cells transfected with the wild-type (“WT”) or the miR-941 mutants (see Fig. 1), were subjected to OGDR stimulation. Results show that transfection of the WT-miR-941 potently inhibited OGDR-induced cytotoxicity in T-HESC cells (Fig. 2g and h), while the mutants were completely ineffective (Fig. 2G and H).

In the primary human endometrial cells OGDR-induced ROS production (tested by superoxide accumulation, Fig. 2i), mitochondrial depolarization (Fig. 2j) and cytosol cytochrome C release (Fig. 2k) were significantly attenuated by ectopic miR-941 overexpression. The latter also alleviated OGDR-induced cytotoxicity in the human endometrial cells (Fig. 2l). These results show that miR-941 overexpression inhibited OGDR-induced oxidative stress, programmed necrosis and cytotoxicity in human endometrial cells.

### miR-941 inhibition upregulates Keap1, suppressing Nrf2 signaling in human endometrial cells

**Fig. 3:**
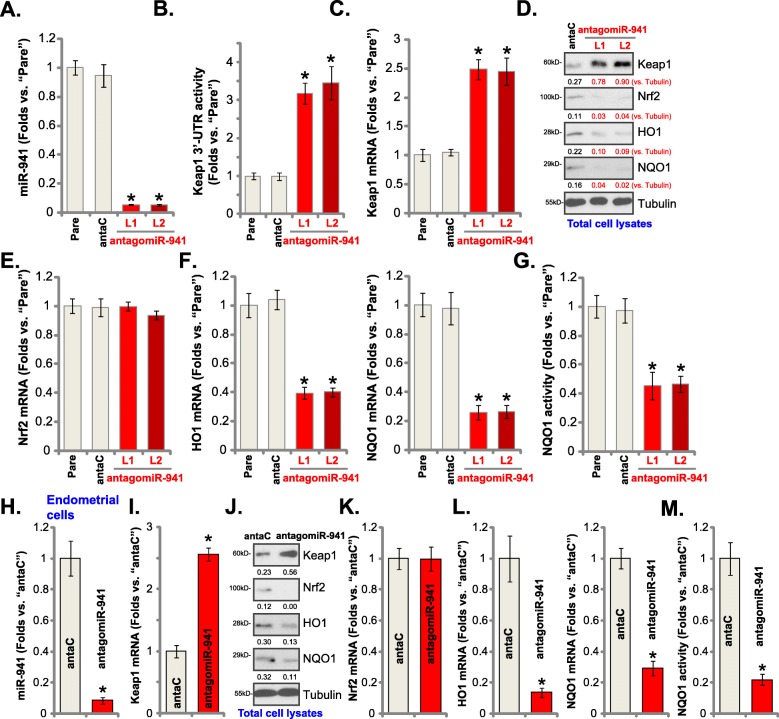
miR-941 inhibition upregulates Keap1, suppressing Nrf2 signaling in human endometrial cells. T-HESC cells were transduced with the pre-microRNA-941 anti-sense lentivirus (“antagomiR-941”), with selection by puromycin the stable cells were established, with control cells transduced with microRNA anti-sense control lentivirus (“antaC”); Expression of mature miR-941 and listed mRNAs were tested by qPCR assays (**a**, **c**, **e** and **f**); Keap1 3′-UTR activity was shown (**b**), with expression of listed proteins in total cell lysates (**d**) tested by Western blotting; The relative NQO1 activity was tested as well (**g**). The primary human endometrial cells were infected with antagomiR-941 or antaC, with expression of listed genes tested by qPCR (**h, i, k**, and **l**) and Western blotting (**j**) assays. The relative NQO1 activity was tested as well (**m**). Expression of listed protein was quantified and normalized (**d** and **j**). Data were presented as mean ± SD (*n* = 5), and results were normalized. * ***P*** < 0.05 vs. “antaC” cells. Experiments in this figure were repeated five times with similar results obtained

To suppress miR-941 expression a pre-microRNA-941 antisense lentivirus (“antagomiR-941”) was transduced into T-HESC endometrial cells, and two stable cell lines, “L1” and “L2”, established. As compared to control cells transduced with miRNA antisense control lentivirus (“antaC”), mature miR-941 levels decreased by over 90% in the antagomiR-941-expressing T-HESC cells (Fig. 3a). Significantly, miR-941 inhibition led to a 3–4 fold increase of 3′-UTR activity of Keap1 in T-HESC cells (Fig. 3b), resulting in upregulation of *Keap1 mRNA* (Fig. 3c) and protein (Fig. 3d) levels. The antagomiR-941 did not affect *Nrf2 mRNA* levels (Fig. 3e), but did decrease Nrf2 protein levels (Fig. 3d). Furthermore, mRNA (Fig. 3f) and protein (Fig. 3d) expression of HO1-NQO1 as well as the NQO1 activity (Fig. 3g) were significantly decreased in T-HESC cells with antagomiR-941. These results suggest that miR-941 is normally involved in the regulation of Keap1 and Nrf2 signaling in T-HESC endometrial cells.

In the primary human endometrial cells antagomiR-941 infection similarly downregulated mature miR-941 expression (Fig. 3h), leading to an increase in Keap1 mRNA (Fig. 3i) and protein (Fig. 3j) levels. AntagomiR-941 inhibited Nrf2 signaling in the primary human endometrial cells, evidenced by decreased expression of Nrf2 protein (but not *Nrf2 mRNA*) (Fig. 3j and k), downregulation of HO1-NQO1 (Fig. 3j and l), and reduced NQO1 activity (Fig. 3m). Therefore, miR-941 inhibition upregulates Keap1 but inhibits Nrf2 signaling in primary human endometrial cells.

### miR-941 inhibition intensifies OGDR-induced programmed necrosis in human endometrial cells

**Fig. 4:**
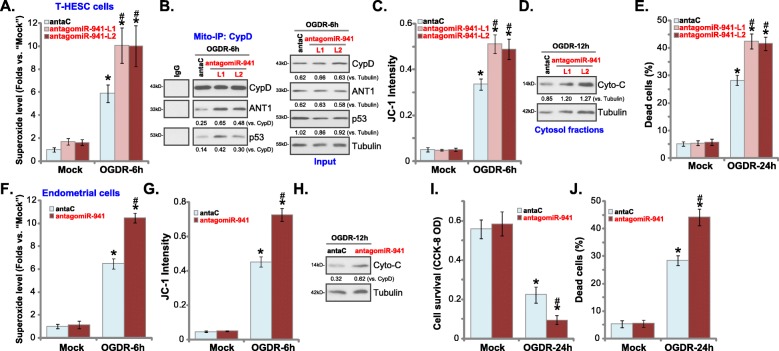
miR-941 inhibition intensifies OGDR-induced programmed necrosis in human endometrial cells. The stable T-HESC cells with the pre-microRNA-941 anti-sense lentivirus (“antagomiR-941”, two lines, “L1/L2”) or microRNA anti-sense control lentivirus (“antaC”) were subjected to OGD exposure for 4 h, followed by re-oxygenation (“OGDR”) for applied time periods, ROS production (superoxide contents, **a**), mitochondrial CypD-ANT1-p53 association (tested by mito-IP assay, **b**) as well as mitochondrial depolarization (JC-1 green fluorescence accumulation, **c**) and cytochrome C release (**d**, testing cytosol proteins) were tested; Cell necrosis was tested by medium LDH release assay (**e**). The primary human endometrial cells were infected with antagomiR-941 or antaC for 48 h, afterwards cells were subjected to the same OGDR stimulation and cultured for applied time periods, ROS production (**f**), mitochondrial depolarization (**g**) and cytosol cytochrome C release (**h**) were tested similarly, with cell survival and necrosis tested by CCK-8 (**i**) and LDH release (**j**) assays, respectively. For the mito-IP assay, CypD-bound ANT1 and p53 were quantified (**b**), with expression of CypD, ANT1 and p53 tested in the “Input” controls (**b**). For the cytochrome C release assay, relative cytosol cytochrome C level was quantified (**d** and **h**). Data were presented as mean ± SD (*n* = 5), and results were normalized. * ***P*** < 0.05 vs. “Mock” treatment in “antaC” cells. ^**#**^***P*** < 0.05 vs. OGDR treatment in “antaC” cells. Experiments in this figure were repeated five times with similar results obtained

As miR-941 inhibition suppresses Nrf2 signaling in human endometrial cells, it could intensify OGDR-induced cytotoxicity. To verify this hypothesis, antagomiR-941-expressing T-HESC cells (Fig. 3) were subjected to OGDR stimulation. As compared to antaC control cells (see Fig. 3), OGDR-induced ROS production tested by superoxide accumulation was significantly increased in antagomiR-941 T-HESC cells (Fig. 4a). Furthermore, OGDR-induced mitochondrial CypD-ANT1-p53 association (Fig. 4b), mitochondrial depolarization (JC-1 green fluorescence accumulation, Fig. 4c) and cytochrome c release to cytosol (Fig. 4d) were augmented bymiR-941 inhibition. Consequently, antagomiR-941 potentiated OGDR-induced necrosis (LDH release to the medium, Fig. 4e) in T-HESC cells. In the primary human endometrial cells, miR-941 inhibition by antagomiR-941 similarly enhanced OGDR-induced ROS production (Fig. 4f), mitochondrial depolarization (JC-1 green fluorescence accumulation, Fig. 4g) and cytochrome C release to cytosol (Fig. 4h). Furthermore, OGDR-induced reduced viability (Fig. 4i) and necrosis (Fig. 4j) were exacerbated with miR-941 inhibition in primary endometrial cells. These results demonstrate that miR-941 inhibition intensifies OGDR-induced cytotoxicity in human endometrial cells.

### miR-941 fails to affect OGDR-induced cytotoxicity in Keap1/Nrf2-KO human endometrial cells

**Fig. 5:**
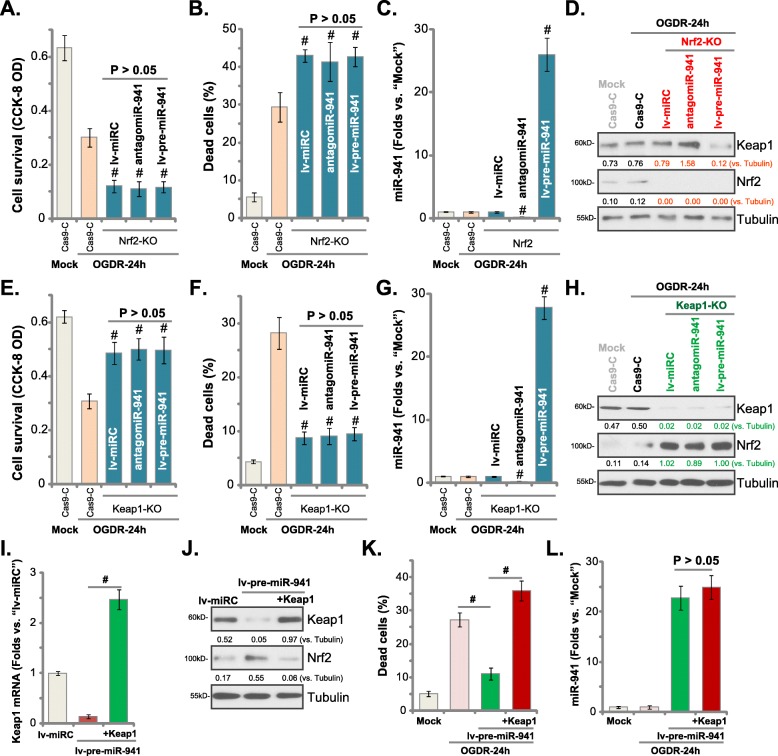
miR-941 fails to affect OGDR-induced cytotoxicity in Keap1/Nrf2-KO human endometrial cells. Stable T-HESC cells with the CRISPR/Cas9-Nrf2-KO construct (“Nrf2-KO”) (**a**-**d**) or the CRISPR/Cas9-Keap1-KO construct (“Keap1-KO”) (**e**-**h**) were further infected with the pre-microRNA-941 anti-sense lentivirus (“antagomiR-941”), the pre-microRNA-941 lentivirus (“lv-pre-miR-941”) or non-sense microRNA lentivirus (“lv-miRC”) for 48 h. These cells or the control cells with empty CRISPR/Cas9-KO construct (“Cas9-C”) were treated with OGD for 4 h, followed by re-oxygenation (“OGDR”) for 24 h, cell survival (**a** and **e**) and necrosis (**b** and **f**) were tested by CCK-8 and LDH release assays, respectively. Expression of mature miR-941 (**c** and **g**) and listed proteins (in total cell lysates, **d** and **h**) were shown. The lv-pre-miR-941-expressing T-HESC cells were further transfected with the UTR-depleted Keap1 construct (“+Keap1”) for 48 h, expression of listed genes was shown (**i** and **j**); Cells were subjected to the same OGDR stimulation for 24 h, cell necrosis (testing medium LDH release, **k**) and mature miR-941 expression (**l**) were tested similarly. Keap1 and Nrf2 protein expression was quantified and normalized to Tubulin (**d**, **h** and **j**). Data were presented as mean ± SD (*n* = 5), and results were normalized.. ^**#**^***P*** < 0.05 vs. OGDR treatment in “Cas9-C” cells (**a**-**c**, **e**-**g**). ^**#**^***P*** < 0.05 (**i** and **k**). Experiments in this figure were repeated three times with similar results obtained

We tested whether Nrf2 activation is essential for miR-941-induced endometrial cell protection against OGDR. Nrf2 knockout (Nrf-KO) T-HESC stable cells were established using a CRISPR/Cas9-Nrf2-KO construct [31], and Nrf2 depletion confirmed by Western blotting (Fig. 5d). Compared to Cas9 control cells (“Cas9-C”), Nrf2-KO cells were more sensitive to OGDR stimulation, showing decreased viability (Fig. 5a) and enhanced cell necrosis (Fig. 5b). Significantly, exogenously altering miR-941 expression, via lv-pre-miR-941 or antagomiR-941 (see qPCR results in Fig. 5c), failed to affect OGDR-induced cytotoxicity in Nrf2-KO T-HESC cells (Fig. 5a and b). Nrf2 KO did not affect Keap1 regulation by lv-pre-miR-941 or antagomiR-941, as shown in Fig. 5d. These results suggest that miR-941-induced endometrial cell protection against OGDR requires Nrf2 signaling.

If targeting and silencing Keap1 is the primary mechanism of miR-941-induced endometrial cell protection against OGDR, Keap1 depletion should mimic miR-941 actions. To test this hypothesis, the CRISPR-Cas9 method was applied to knockout Keap1 in T-HESC cells (using a previously described protocol [31]). OGDR-induced cytotoxicity (Fig. 5e) and necrosis (Fig. 5f) were largely attenuated in Keap1-KO T-HESC cells (vs. Cas9-C cells), mimicking the actions of miR-941 overexpression. Importantly, in the Keap1-KO cells, lv-pre-miR-941 and antagomiR-941 were both ineffective against OGDR-induced actions (Fig. 5e and f), although both altered miR-941 expression (Fig. 5g). As expected (Fig. 5h), Keap1 depletion in the Keap1-KO T-HESC cells resulted in significant Nrf2 protein accumulation.

To further support our hypothesis, an UTR-deleted Keap1 construct was transfected into lv-pre-miR-941-expressing T-HESC cells, resulting in *Keap1 mRNA* (Fig. 5i) and protein (Fig. 5j) re-expression, but Nrf2 protein reduction (Fig. 5j). Functional studies show that re-expression of Keap1 reversed the lv-pre-miR-941-induced T-HESC cell protection against OGDR, leading to cell necrosis (Fig. 5k). Notably, Keap1 re-expression did not affect miR-941 overexpression in lv-pre-miR-941-T-HESC cells (Fig. 5l). These results further support that Keap1-Nrf2 cascade activation is essential for miR-941-induced endometrial cell protection against OGDR.

## Discussion

The expression and potential function of miR-941 in human endometrial cells is unknown. The study by Hu et al.*,* has shown that miR-941 is a human-specific miRNA, highly expressed in the human brain and in other human cells [56]. It has regulatory effects on gene expression, participating in hedgehog- and insulin-signaling pathways [56]. Zhang et al.*,* have shown that miR-941 levels are significantly downregulated in hepatocellular carcinoma (HCC) tissues. Functionally, miR-941 negatively regulated KDM6B (lysine (K)-specific demethylase 6B) to inhibit HCC cell epithelial-mesenchymal transition (EMT) and cell migration/invasion [57]. Bai et al.*,* reported that miR-941 levels are significantly higher in acute coronary syndrome patients than those in healthy controls [58].

Recent studies demonstrate that miRNA-induced silencing of Keap1, the Nrf2 suppressor protein, is a novel strategy to regulate Nrf2activation in human cells. In breast cancer cells miR-200a targeted Keap1 to activate Nrf2 signaling cascade [36]. In SH-SY5Y neuroblastoma cells and differentiated human neural progenitor cells, miR-7 activated Nrf2 signaling by targeting and silencing Keap1 [35]. In retinal pigment epithelium cells (RPEs) and retinal ganglion cells (RGCs), miR-141 activated Nrf2 signaling by targeting miR-141, protecting cells against ultra-violet (UV)-induced oxidative stress [59]. A recent study by Xu et al.*,* has shown that Keap1-targeting miR-626 activated Nrf2 signaling and protected RPEs from oxidative injury [31]. Therefore, Keap1-targeting miRNAsinduceNrf2 activation to protect human cells from oxidative stress.

The results of this study confirm that miR-941 is a direct and specific Keap1-targeting miRNA, that regulates the Keap1-Nrf2 cascade in human endometrial cells. In T-HESC cells and primary human endometrial cells forced miR-941 overexpression inhibited Keap1 3′-UTR reporter luciferase activity and downregulated Keap1 mRNA/protein expression, subsequently leading to Nrf2 activation. Conversely, Keap1 3′-UTR reporter luciferase activity and expression were elevated in endometrial cells with miR-941 inhibition, whereas Nrf2 activation was inhibited. RNA-Pull down experiments confirmed that miR-941 directly binds Keap1 mRNA in T-HESC cells. Functional studies confirmed that miR-941 exerted significant endometrial cytoprotection against OGDR. Ectopic miR-941 overexpression in endometrial cells largely inhibited OGDR-induced ROS production and programmed necrosis. Thus, miR-941 promotes Nrf2 cascade activation by targeting and silencing Keap1. Therefore, miR-941 expression provides a novel strategy to protect human endometrial cells from OGDR and possible ischemia-reperfusion injuries.

Our results support that activation of the Keap1-Nrf2 cascade is absolutely required for miR-941-induced endometrial cell protection. Nrf2 KO, using the CRISPR/Cas9 method, abolished miR-941-induced endometrial cytoprotection against OGDR. Furthermore, CRISPR/Cas9-induced Keap1 KO mimicked miR-941 actions and largely attenuated OGDR-induced cytotoxicity in T-HESC cells. Ectopic miR-941 overexpression and miR-941 inhibition were both ineffective against OGDR in the Nrf2-KO and Keap1-KO T-HESC cells. Significantly, restoring Keap1 expression, using an UTR-depleted Keap1 construct, abolished miR-941-induced endometrial cytoprotection against OGDR. Furthermore, transfection of the miR-941 mimics, with mutations at the binding sites to Keap1’s 3′-UTR, failed to protect endometrial cells from OGDR. These results demonstrate that in endometrial cells miR-941-induced activation of the Keap1-Nrf2 cascade is responsible for protection against OGDR.

## Conclusion

MiR-941 negatively regulates Keap1 to activate Nrf2 signaling cascade, thus protecting human endometrial cells from OGDR-induced oxidative stress and programmed necrosis.

## Data Availability

All data generated during this study are included in this published article. Data will be made available upon request.

## Abbreviations

ANT1: Adenine nucleotide translocator type 1
ARE: Antioxidant responsive element
CCK-8: Cell counting kit-8
CypD: Cyclophilin-D
GCLC: γ-glutamyl cysteine ligase catalytic subunit
GRh2: Ginseng Rh2
HO-1: Heme oxygenase-1
Keap1: Kelch-like ECH-associated protein 1
KGF: Keratinocyte growth factor
KO: Knockout
LDH: Lactate dehydrogenase
miR-941: MicroRNA-941
miRNA: MicroRNA
Mito-IP: Mitochondrial immunoprecipitation
mPTP: Mitochondria permeability transition pore
ncRNAs: Non-coding RNAs
NQO1: NAD(P)H:quinone oxidoreductase 1
Nrf2: Nuclear-factor-E2-related factor 2
OD: Optic density
OGD: Oxygen and glucose deprivation
OGDR: OGD-re-oxygenation
qPCR: Quantitative real time-PCR
SD: Standard deviation
UTR: Untranslated region
